# Effect of Impulsive Compression Treatment on Postoperative Complications After Open Peripheral Vascular Revascularization (In Situ): Protocol for a Randomized Control Trial

**DOI:** 10.2196/resprot.8799

**Published:** 2018-02-20

**Authors:** Tenna Klit, Marie Dahl, Kim Christian Houlind, Hans Ravn

**Affiliations:** ^1^ Department of Vascular Surgery Hospital Lillebaelt University of Southern Denmark Kolding Denmark; ^2^ Department of Vascular Surgery Viborg Hospital Viborg Denmark

**Keywords:** Critical leg ischemia, postoperative edema, Foot pump

## Abstract

**Background:**

In patients with critical leg ischemia (CLI), the standard operative choice is an in situ bypass to the lower extremity to improve the patients´ prognosis and quality of life. Postoperative complications after surgery occur in 18 % of the patients, prolonging hospitalization and convalescence. The main operative complication is edema. This can be prevented by early mobilization or stimulation of the natural venous pump in the leg.

**Objective:**

To investigate whether compression therapy with foot pump reduces postoperative edema, facilitates wound healing of the operation wounds, promotes healing of ischemic ulcers, and shortens hospitalization, increasing and improving the patient’s subjective quality of life faster.

**Methods:**

The protocol is designed as a randomized, unblinded prospective study with 50 patients in each group. Standard postoperative routines after bypass surgery, including short-stretch bandaging of the operated legs, are compared to supportive stimulation of the venous pump by an impulsive compression foot pump. The postoperative edema of the leg is measured 10 cm below the patella and 8 cm proximal to the medial malleolus. Measurements are performed preoperatively, 1 and 4 days postoperatively and at discharge.

**Results:**

The primary endpoint is reduction of leg edema by at least 50%. The secondary endpoint includes earlier mobilization in the pump group and decreased length of stay in hospital. Quality of life is evaluated through the European Health Related Quality of Life Questionnaire 5 Dimensions (EQ-5D) and Vascular Quality of Life Questionnaire-6 (VascuQol-6) questionnaires. The start of the study is February 1, 2018, and the end of the study is February 1, 2020. First results will be available April 2020.

**Conclusions:**

In orthopedic surgery of the lower extremities, the use of foot pumps has shown a reduction of edema and thrombosis in risk patients. Although important positive effects may be expected after vein bypass surgery, no reports have yet investigated the use of the device in vascular-operated patients and no analysis or meta Cochrane reviews are available in this field.

**Trial Registration:**

ClinicalTrials.gov NCT03192982; https://clinicaltrials.gov/ct2/show/NCT03192982 (Archived by WebCite at http://www.webcitation.org/6xMZJ06dw)

## Introduction

Critical leg ischemia (CLI) is a predominant cause of poor wound healing, leg amputation [1], and has a significant, negative impact on quality of life [2]. In patients with CLI, the standard operative choice is an in situ bypass to the lower extremity to improve the patient's prognosis and quality of life. However, postoperative complications after an in situ bypass occur in 18% of patients, prolonging their hospitalization and convalescence. The main postoperative complication is edema. Measured 10 cm below the patella, edema is developed among approximately 6%-8% during the first day, increasing to 8%-10% at the fourth day (nonpublished). Postoperative edema can be prevented by early mobilization which stimulates the natural venous pump in the leg, but mobilization is often difficult in patients with CLI due to wound problems prior to surgery. In addition, CLI is associated with age and limited walking distance and coexists with other manifest diseases, which might also make mobilization difficult. Supportive stimulation of the venous pump by an impulsive compression foot pump may be an effective solution. Patient’s stimulation of the venous plexus in the arch of the foot has shown to reduce leg edema and thrombosis risk in patients undergoing orthopedic surgery on the lower extremities [3-10]. However, in the literature, there is no description of treatment of vascular surgical patients treated with foot pump. Thus, the primary objective of this study is to investigate whether a foot pump is effective in reducing postoperative edema in patients undergoing an in situ bypass.

### Background and Rationale

In situ bypass to the lower extremity is the standard operation choice on patients with critical leg ischemia, accounting for approximately 18% of patients’ wound problems [11], prolonging their hospitalization time and convalescence. One of the reasons for wound problems is postoperative edemaThe edema is expected after open vascular reconstruction (in situ bypass surgery). Early mobilization can prevent development of the postoperative edema. Through mobilization, the patient stimulates the natural venous pump in the leg. However, patients are often hard to mobilize in an early stage due to the wound and age. Stimulation of the venous plexus in the arch of the foot has, in other categories of patients, shown to reduce postoperative edema of leg and thrombosis risk in patients undergoing orthopedic surgery on the lower extremities. The subjective quality of life is often impaired in relation to the above mentioned surgical treatment. Through a quicker recovery, the patient can rapidly regain mobility and walking capability.

There is no analysis or meta Cochrane reviews in this field.

### Objectives

The objectives will be as follows:

1. To investigate whether compression therapy with foot pump reduces postoperative edema.
2. Through reducing edema, facilitate wound healing of the operation wounds, promote healing of ischemic ulcers, and decrease major amputation rate.
3. Shorten hospitalization.
4. Increase and improve the patient’s subjective quality of life faster.

## Methods

The trial design is a 1:1 randomized prospective study. All surgical procedures will be performed by vascular surgeons. The postoperative care will be performed by special nurses in cooperation with vascular surgeons at the Department of Vascular Surgery, Lillebaelt Hospital, Denmark.

### Participants, Interventions, and Outcomes

#### Eligibility criteria

##### Inclusion:

The study will include patients who have undergone in situ from common femoral artery to a popliteal artery above knee/below knee or crural artery.

##### Exclusion:

The study will exclude patients with former deep vein thrombosis, symptomatic postthrombotic syndrome, or ischemic wounds that are in such manner that compression of the foot is not possible.

##### Age of Subjects:

Subjects will be between the ages of 18 and 99 years.

### Interventions

Post operation, the foot pump will be placed on the foot, according to randomization. The pump must be placed on the foot immediately after the operation is finished. The foot pump will be left on the foot until full mobilization is reached. The pump should not harm or hurt the patient when it is placed on the foot in the correct position.

The control group will follow the department’s ordinary postoperative routines for inset bypass short-stretch bandaging. The short-stretch bandage is a padded bandage that stretches from the toes and up to the upper thigh with a 40 mmHg pressure.

### Outcomes

#### End points

The end points are as follows:

1. Reduction of leg edema by 50%, 10 cm below the patella and 8 cm proximal of the medial malleolus on the operated leg three days post operation in patients treated with impulsive compression in comparison to nontreated.
2. “Wound complications” are defined according to the national Danish vascular registry “karbase”, including infections, hematoma, and lymph excretion over two days. The pump is expected to have influence on the wounds on the thigh/crus and not in the groin. We therefore separate on wound complications in the groin and the other part of the leg.
3. Time to mobilization in pump treated patients (ie, patient can get out of bed and go to the toilet).
4. Length of stay in hospital.

### Participant timeline

#### Trial baseline

The start of patient inclusion is February 1, 2018, and the study will be finished February 28, 2020. The first results are expected by April 30, 2020.

#### Time Schedule for Measuring Assessing Symptoms As Edema

The time schedule will be as follows:

1. In the open clinic when the patient is included.
2. The day after the operation.
3. Day 4 and the day of patient discharge.
4. Day 42 (control in our open clinic for control of the operation results).

The measure procedure will be as follows:

1. The circumference of the leg will be measured at 10 cm below the distal part of the patellae and 8 cm above medial malleolus.
2. In the same procedure, we will evaluate pain by the Visual Analogue Scale (VAS).
3. Randomization when the patient is on the operation table.
4. Operation performed.
5. Post operation.
6. The control group follows the department’s ordinary postoperative routines for inset bypass — short-stretch bandage. A short-stretch bandage is a padded bandage that stretches from the toes and up to the upper thigh with a 40 mmHg pressure.
7. Time for full mobilization is recorded in all patients.
8. Reoperations is noted.
9. Toe pressure is measured at inclusion, immediately post operation and at discharge.
10. Time for discharge is noted.
11. Paraclinical factors to be examined: confusion, pneumonia, infection of the urinary tract.
12. Duplex study at Day 42 of reverse flow in the deep veins associated with standard duplex study of AV fistulas postoperatively.
13. Quality of life questionnaire (VascuQol-6) baseline + 42 days + 6-month + 12-month, (Appendix 1).
14. EQ-5D baseline + 42 days + 6-month + 12-month, (Appendix 2).

### Sample size

The sample will comprise of 50 patients in each group with a total of 100 patients.

### Statistical Analysis and Structural Analysis

Through decreasing the postoperative edema by 50 %, we can reduce the wound complication rate for patients from 18% to 12%, given a sigma of 10 and the two-sided test, with an alpha value of 0.05 and beta of 0.80 involving 44 patients in each group. Assuming a dropout rate of 6 patients in each group, the “sample size” will rise to 50 in each group.

### Recruitment

Patients will follow the department’s normal procedure in terms of in situ bypass. They will then be asked in the outpatient’s clinic during a visit to prepare for the operation.

### Assignment of Intervention

#### Allocation

Allocation will be by randomization.

#### Sequence generation

The study will use an online randomization program. The total distribution between the two groups is 1:1, but a block randomizing will be used in different sizes.

#### Allocation concealment

#### Mechanism

Last minute randomization will occur at onset of surgery by study coordinator.

### Implementation

#### Informed consent

Patients with critical leg ischemia that are being assessed for inset bypass will be offered to participate in the trial by ambulatory visit. There will be a sheet with written information handed out and the patients will also receive oral information about the trial. The patient then has the option for consideration until the randomization happens at admission to operation. The oral information will be given by the research nurse and study physician through a phone call from a study room in the outpatient clinic at Department of Vascular Surgery, Lillebaelt Hospital, Denmark. The patient is then given the written information to take home for reading and can have an observer present at the hospital where the patient must submit a statement of participation in the study. The time between ambulatory visit and hospitalization will usually be 7 to 14 days, depending on the severity of the patient’s ischemia.

### Blinding

Patient and surgeon will be blinded to the allocation during surgery but will need to be unblinded at the time when the intervention is initiated immediately after surgery.

### Data Collection, Management, and Analysis

#### Data Collection Methods

##### Data Application Sheet

A data application sheet will be created for all the included patients. The information will be fed into the database for analysis.

#### Data management

Data management will be handled in the Data Analysis and Statistical Software program (STATA version 13). A special data applications model has been constructed for the study in Open (Odense Patient Data Explorative Network).

### Statistical methods

Descriptive statistics will be used to report study results. For binary and categorical variables, tests of differences between the intervention and control group will be analyzed by the chi-square test. For continuous variables, initial tests will be performed to check for normality of data by using histograms, Q-Q plots and the Shapiro-Wilk test. Normally and nonnormally distributed variables will be analyzed by *t* test or the Wilcoxon rank sum test, respectively. *P* values <.05 will be considered statistically significant.

### Monitoring

#### Data Monitoring and Auditing

Data monitoring and auditing will be conducted by yearly report. The ethical committee will receive a list on all serious adverse effects in the study (expected and unexpected) and serious incidents together with a report about the security of the patients.

### Risks, Side Effects and Disadvantages

The foot pump treatment is noninvasive. Patients should not experience aches or pain relating to the treatment method. The pump is used in other European countries on an empirical basis and Communautés Européennes (CE) mark. Furthermore, the Duplex study is done as routine examinations in the department and is noninvasive. The side effects and risks of pump therapy can be considered insignificant.

### Patient Insurance

The subject is covered by the patient compensation scheme in the region of southern Denmark.

## Results

Enrollment will begin February 1, 2018, and enrollment will end February 28, 2020. Results are expected by April 30, 2020.

## Discussion

In orthopedic surgery of the lower extremities, the use of foot pumps has shown a reduction of edema and thrombosis in risk patients. No reports describe vascular-operated patients, and there does not exist analyses or meta Cochrane reviews in this field. Postoperative edema of the leg increases the risk of other complications (operation wound complications, infections, hematoma and lymph excretion), prolongs the hospitalization, and decreases quality of life. This study can change the postoperative procedures of the patients operated for CLI and reduce the cost of the treatment, increasing the quality of life of patients.

## Appendix

Multimedia Appendix 1 VascuQol-6.

Multimedia Appendix 2 Eq-5d.

## Abbreviations

CE: Communautés Européennes
CLI: critical leg ischemia
EQ-5D: European Health Related Quality of Life Questionnaire 5 dimension
Open: Odense Patient Data Explorative Network
STATA: Data Analysis and Statistical Software program
VAS: visual analogue scale
VascuQol-6: Vascular Quality of Life Questionnaire-6
