# Supplementary material for: Effect of Impulsive Compression Treatment on Postoperative Complications After Open Peripheral Vascular Revascularization (In Situ): Protocol for a Randomized Control Trial
Source: JMIR Res Protoc. 2018 Feb 20;7(2):e58. doi: 10.2196/resprot.8799 (PMC5840475; doi:10.2196/resprot.8799)
Supplement: Multimedia Appendix 1 [file resprot_v7i2e58_app1.pdf]

VascuQol – 6 Spørgeskemaet

1. På grund af det dårlige blodomløb i mine ben **er det generelle omfang af aktiviteter, som jeg gerne ville have udført i løbet af de seneste to uger, blevet**…

☐*Alvorligt begrænset—de fleste aktiviteter er ikke blevet udført*
☐*Moderat begrænset - adskillige aktiviteter er ikke blevet udført*
☐*Ganske lidt begrænset—kun ganske få aktiviteter er ikke blevet udført*
☐*Slet ikke begrænset—har udført alle de aktiviteter jeg havde tænkt mig*

2. I løbet af de seneste to uger **har mine ben føltes trætte eller svage**…

☐*Hele tiden*
☐*Lidt af tiden*
☐*Næsten ikke noget af tiden*
☐*Aldrig*

3. I løbet af de seneste to uger **har det dårlige blodomløb i mine ben betydet, at min evne til at gå er blevet**…

☐*Udelukket, jeg har slet ikke kunnet gå*
☐*Meget begrænset*
☐*Lidt begrænset*
☐*Slet ikke begrænset*

4. I løbet af de seneste to uger **har jeg været bekymret over at have dårligt blodomløb i mine ben**…

☐*Hele tiden*
☐*Lidt af tiden*
☐*Næsten ikke noget af tiden*
☐*Aldrig*

5. I løbet af de seneste to uger **har det dårlige blodomløb i mine ben betydet, at min evne til at tage del i sociale aktiviteter har været**…

☐*Udelukket, jeg har slet ikke kunnet udføre denne aktivitet*
☐*Meget begrænset*
☐*Lidt begrænset*
☐*Slet ikke begrænset*

6. I løbet af de seneste to uger **har det, når jeg har haft smerter i benet (eller foden), været årsag til**…

☐*Meget stort ubehag eller fysisk lidelse*
☐*Nogen grad af ubehag eller fysisk lidelse*
☐*Meget lidt ubehag eller fysisk lidelse*
☐*Intet ubehag eller fysisk lidelse*

Ophavsret - Mark Morgan
Alle rettigheder forbeholdes
