# Supplementary material for: Effect of Impulsive Compression Treatment on Postoperative Complications After Open Peripheral Vascular Revascularization (In Situ): Protocol for a Randomized Control Trial
Source: JMIR Res Protoc. 2018 Feb 20;7(2):e58. doi: 10.2196/resprot.8799 (PMC5840475; doi:10.2196/resprot.8799)
Supplement: Multimedia Appendix 2 [file resprot_v7i2e58_app2.pdf]

Helbredsspørgeskema

Dansk version for Danmark

(Danish version for Denmark)

Denmark (Danish) © 2009 EuroQol Group EQ-5D™ is a trade mark of the EuroQol Group
Final Version 1.0 (Denmark - Danish) - 16Jun2015

Visit - ________	Date completed: dd / mm / yyyy	Time completed: 24-hour clock	EQ-5D-5L

Under hver overskrift bedes du sætte kryds i DEN kasse, der bedst beskriver dit helbred I DAG.

BEVÆGELIGHED

Jeg har ingen problemer med at gå omkring ❑

Jeg har lidt problemer med at gå omkring ❑

Jeg har moderate problemer med at gå omkring ❑

Jeg har store problemer med at gå omkring ❑

Jeg kan ikke gå omkring ❑

PERSONLIG PLEJE

Jeg har ingen problemer med at vaske mig eller klæde mig på ❑

Jeg har lidt problemer med at vaske mig eller klæde mig på ❑

Jeg har moderate problemer med at vaske mig eller klæde mig på ❑

Jeg har store problemer med at vaske mig eller klæde mig på ❑

Jeg kan ikke vaske mig eller klæde mig på ❑

SÆDVANLIGE AKTIVITETER *(fx. arbejde, studie, husarbejde, familie- eller fritidsaktiviteter)*

Jeg har ingen problemer med at udføre mine sædvanlige aktiviteter ❑

Jeg har lidt problemer med at udføre mine sædvanlige aktiviteter ❑

Jeg har moderate problemer med at udføre mine sædvanlige aktiviteter ❑

Jeg har store problemer med at udføre mine sædvanlige aktiviteter ❑

Jeg kan ikke udføre mine sædvanlige aktiviteter ❑

SMERTER / UBEHAG

Jeg har ingen smerter eller ubehag ❑

Jeg har lidt smerter eller ubehag ❑

Jeg har moderate smerter eller ubehag ❑

Jeg har stærke smerter eller ubehag ❑

Jeg har ekstreme smerter eller ubehag ❑

ANGST / DEPRESSION

Jeg er ikke ængstelig eller deprimeret ❑

Jeg er lidt ængstelig eller deprimeret ❑

Jeg er moderat ængstelig eller deprimeret ❑

Jeg er meget ængstelig eller deprimeret ❑

Jeg er ekstremt ængstelig eller deprimeret ❑

Denmark (Danish) © 2009 EuroQol Group EQ-5D™ is a trade mark of the EuroQol Group
Final Version 1.0 (Denmark - Danish) - 16Jun2015

Visit - ___________ | Date completed: __ __ (dd) __ __ (mm) ____ (yyyy) | Time completed: __ __ __ __ (24-hour clock) | **EQ-5D-5L**

- Vi vil gerne vide, hvor godt eller dårligt dit helbred er I DAG.
- Denne skala er nummereret fra 0 til 100.
- 100 svarer til det bedste helbred, du kan forestille dig.
  0 svarer til det dårligste helbred, du kan forestille dig.
- Sæt et X på det sted på skalaen, der viser, hvordan dit helbred er I DAG.
- Skriv derefter det tal, du har markeret på skalaen, ind i boksen nedenunder.

DIT HELBRED I DAG = ☐

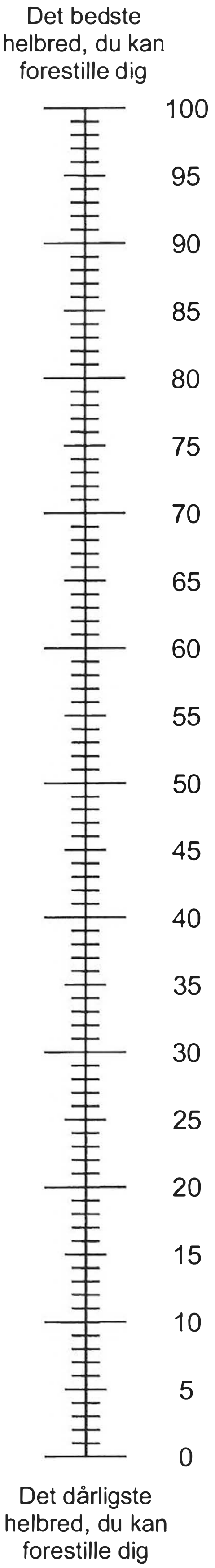

Denmark (Danish) © 2009 EuroQol Group EQ-5D™ is a trade mark of the EuroQol Group
